# Clinical Implications of Geometric and Dosimetric Uncertainties of Inter- and Intra-Fractional Movement during Volumetric Modulated Arc Therapy for Breast Cancer Patients

**DOI:** 10.3390/cancers13071651

**Published:** 2021-04-01

**Authors:** Jason Joon Bock Lee, Ik Jae Lee, Yeonho Choi, Mi Jin Jeon, Il Hun Jung, Ho Lee

**Affiliations:** 1 Department of Radiation Oncology, Gangnam Severance Hospital, Yonsei University College of Medicine, Seoul 06273, Korea; JBWOLF86@yuhs.ac (J.J.B.L.); IKJAE412@yuhs.ac (I.J.L.); YUNHOC12@yuhs.ac (Y.C.); OLIVE825@yuhs.ac (M.J.J.); JLH1000@yuhs.ac (I.H.J.); 2 Department of Radiation Oncology, Kangbuk Samsung Hospital, Sungkyunkwan University School of Medicine, Seoul 03181, Korea

**Keywords:** cone-beam computed tomography, image-guided radiotherapy, volumetric modulated arc therapy, breast cancer, intrafraction motion

## Abstract

**Simple Summary:**

Radiotherapy is an essential treatment modality for breast cancer. Compared to conventional radiotherapy techniques, modern radiotherapy with fewer fractions and smaller target volumes requires higher accuracy. Image-guidance using cone-beam computed tomography (CBCT) is one of the most common methods used for positional verification before treatment. This study reports geometric and dosimetric outcomes evaluated by analyzing CBCT images acquired before and during treatments. The positional change and internal movement of the patient were less than 1 cm in most cases without significant deviation in the dosimetric parameters of interest. However, there were cases involving extreme variation, which resulted in insufficient radiation delivered to the target areas and increased radiation exposure to adjacent normal organs. The results of the current study suggest that unexpected intra-fractional motion may occur, prompting for marginal adaptation in selected patients who are deemed to suffer from this kind of event.

**Abstract:**

With the introduction of modern sophisticated radiotherapy (RT) techniques, the significance of accuracy has increased considerably. This study evaluated the necessity of pre-treatment and intra-fractional cone-beam computed tomography (CBCT) by analyzing inter- and intra-fractional CBCT images of breast cancer patients receiving RT. From 57 patients, 1206 pre-treatment CBCT and 1067 intra-fractional CBCT images were collected. Geometric movements of patients were measured quantitively in both inter- and intra-fractional CBCT, and changes in dosimetric parameters were evaluated in selected patients with extreme intra-fractional movement. For right-sided breast cancer patients, left-sided breast cancer patients treated using deep-inspiration breath hold (DIBH), and left-sided breast cancer patients treated using continuous positive airway pressure (CPAP), median inter-fractional deviations were 0.53 (range 0.06–2.98) cm, 0.66 (range 0.08–4.41) cm, and 0.69 (range 0.04–3.80) cm, and median intra-fractional deviations were 0.14 (range 0.00–0.62) cm, 0.23 (range 0.02–0.96) cm, and 0.24 (0.00–1.15) cm, respectively. Modified plans reflecting large changes in intra-fractional position in 10 selected cases revealed insufficient target coverage in seven cases and more than 20-fold increase in the volume of heart receiving at least 25 Gy in two cases. Intra-fractional verification, as well as pre-treatment verification, might be considered in patients using DIBH or CPAP.

## 1. Introduction

As modern radiation therapy (RT) with highly sophisticated techniques becomes more widely used in treating cancer patients [1], ensuring RT accuracy is becoming more important than ever. Prominent characteristics of modern RT include increasing frequency of intensity-modulated RT (IMRT) and stereotactic body RT (SBRT) with image-guidance. In clinical practice, volumetric imaging modalities such as cone-beam computed tomography (CBCT) and mega-voltage computed tomography are frequently used for pre-treatment verifications [2]. IMRT shows benefits in target coverage with normal organ sparing, whereas SBRT is characterized by very high dose delivery in a small number of fractions to a restricted target area. However, the treatment time for each fraction increases due to pre-treatment verification and the utilization of a complex treatment plan with multi-directional beams. Longer treatment times may lead to extensive patient movement during RT, which may alter the quality of actual RT delivery to the target area. To this end, many studies have evaluated the effects of intra-fractional movement on dosimetric outcomes in various organs and regions of the body, including prostate [3,4], lung [5,6], head and neck, and abdomino-pelvic areas [7].

Conventionally, RT has been one of the most common treatment modalities used when managing breast cancer. Despite its lengthy history of utilization, the current trends in RT for breast cancer have been changing drastically. For example, there are wider implications for hypofractionation [8,9] and the application of partial breast irradiation (PBI) in selected patients with small target volumes [9,10]. Moreover, techniques such as deep-inspiration breath hold (DIBH) and continuous positive airway pressure (CPAP) may avoid heart toxicity when treating left-sided breast cancer. However, these techniques may be physically challenging for some patients and may lead to a higher incidence of inter- and intra-fractional positional error. To our best knowledge, studies regarding the measurement of intra-fractional error in breast cancer patients with CBCT images are not plenty. Therefore, this study evaluated the plausibility of employing not only inter-fractional but also intra-fractional CBCT in clinical practice by analyzing inter- and intra-fractional CBCT images of breast cancer patients with respect to positional changes and subsequent dosimetric alterations.

## 2. Materials and Methods

### 2.1. Patients

The intra-fractional CBCT images of 57 patients treated in Gangnam Severance Hospital from June 2019 to November 2020 were collected. All patients underwent non-contrast CT simulation in a supine position with a customized arm support using a breast board. All patients received single, partial-arc volumetric modulated arc therapy (VMAT) with pre-treatment verification using kV-CBCT. All VMAT plans used 6 MV coplanar photon beams with a single isocenter for each patient and were designed using the RayStation treatment planning system (TPS) (RaySearch Laboratories AB, Stockholm, Sweden). The Versa HD^TM^ linear accelerator (Elekta Limited, Stockholm, Sweden) equipped with a multi-leaf collimator was used for RT. To spare critical organs at risk (OARs), patients with left-sided breast cancer underwent either DIBH or CPAP to inflate the lungs and increase the distance from the chest wall to the heart and coronary arteries. Patients with right-sided breast cancer were treated without these particular techniques. The dose scheme for RT was either the conventional regimen of 50.4 Gy for patients who received regional node irradiation or a hypofractionated regimen of 40.05 Gy in 15 fractions for patients receiving whole breast irradiation only. A bolus was applied for the initial 14 fractions to patients who received radical mastectomy with reconstruction and was removed for the remaining 14 fractions to avoid excessive skin toxicity.

### 2.2. CBCT Data Acquisition

CBCT images were acquired daily at two time points: one before the treatment and another during RT. After positioning the patient according to the three skin markers drawn during simulation, pre-treatment CBCT images were acquired. For each fraction, pre-treatment CBCT images were registered to simulation CT images by an automatic configuration process followed by fine manual adjustment, if necessary, by the physician or therapist. During this configuration process, the chest wall and breast were the primary points of interest while other reference structures such as bony spines were evaluated as well to achieve an ideal treatment setup. In rare cases showing significant dislocation of the breast even if the chest wall matched well, a treating physician decided the course of action among several options which included continuing the treatment, repositioning the patient, and conducting an adaptive CT simulation. All these corrections were executed by mechanically adjusting the couch. During partial-arc VMAT, a simultaneous intra-fractional CBCT scan was taken as the gantry rotated around the patient. The gantry rotation angle (the initial position of the gantry indicated a benchmark of 0°) for intra-fractional CBCT acquisition ranged from −60° to 160° clockwise for left-sided breast cancer and −160° to 60° counterclockwise for right-sided breast cancer. A small field-of-view protocol using an S20 collimator and F0 filter was utilized (120 kVp, 20 mA, and a 20 ms time period for each projection).

### 2.3. Data Analysis

**Figure 1:**
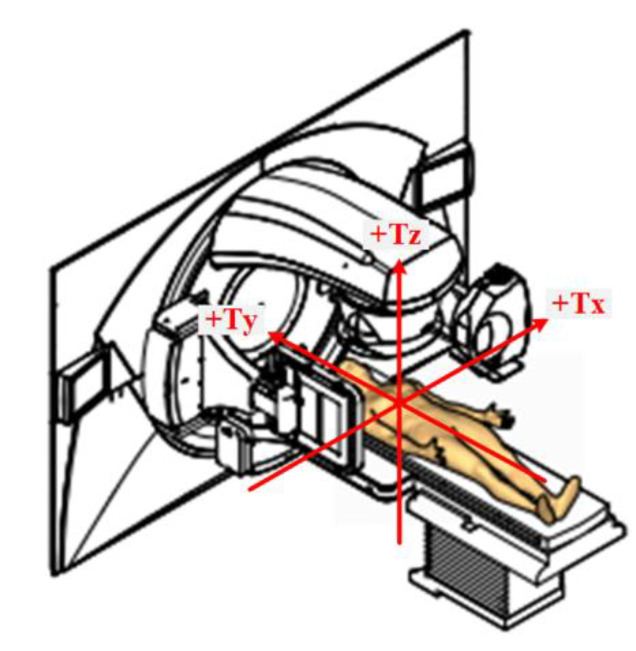
Schematic description of patient set-up during radiotherapy and measurement method. Each arrow shows the direction (plane) of measurement. The difference between the position of the patient during simulation and the actual position of the patient during radiotherapy, as depicted by the gold-colored body, was calculated by comparing simulation CT images with intra-fractional CBCT images.

The change in position between CT simulation and the actual position of the patient before and during RT was assessed by pre-treatment CBCT and intra-fractional CBCT, respectively, according to the differences in x-, y-, and z-axes as shown in Figure 1. After the difference in each axis was measured, the absolute distance between two CT images was measured by the formula:

$$\sqrt{\Delta x^{2}+\;\Delta y^{2}+\Delta z^{2}}.$$

The configuration process for intra-fractional CBCT was identical to pre-treatment CBCT and was monitored by an experienced radiation oncologist and physicist. The chest wall was used as the baseline standard for configuration, and the bony spine was used as a supplemental benchmark. Ten fractions which showed the biggest deviations were selected. From them, we created a virtual RT plan according to the measured differences between the original CT simulation and the intra-fractional CBCT scan. New dose-volume histograms (DVH) were acquired for the modified plan. All target volumes and normal organs were contoured using MIM software version 6.6.14 (MIM Software, Inc., Cleveland, OH, USA), and the DVHs for each plan were generated by RayStation TPS. OARs were delineated by either an experienced radiation oncologist or dosimetrist. Clinical target volume of breast or chest wall, and regional lymph nodes if necessary depending on treating physician’s discretion, was contoured based on ESTRO consensus guideline [11]. Uniform set-up margin of 2–3 mm, mostly 2 mm, was added to construct planning target volume (PTV). Doses to the PTV, the lungs, and the heart were critical points of interest in this study. To evaluate precise dose coverage and to avoid normal organs, the following DVH values were collected: D_95% of the PTV_, V_95% of the prescription dose_, mean lung dose, V_5 Gy_ of ipsilateral lung, V_20 Gy_ of the ipsilateral lung, D_0.2 cc_ for the heart, and V_25 Gy_ of the heart. D_”volume”_ indicated the cumulative dose to the most exposed “volume,” and V_”dose”_ was defined as the percentage of the total volume receiving a dose that exceeded the “dose.” At our institution, the target coverage of a treatment plan was regarded ideal if both D_95% of the PTV_ and V_95% of the prescription dose_ exceeded 95% of the prescription dose and 95% of the PTV, respectively. The treatment plan was regarded as acceptable if these values were within the 90–95% range, and it was regarded as unacceptable if one of these values was below 90%. A comparison of the extent of positional change from pre-treatment CBCT to intra-fractional CBCT for each fraction was performed using the Wilcoxon signed-rank test. The Kruskal–Wallis test and a subsequent Mann–Whitney U test were used to compare the extent of movement according to location and RT technique. *p*-values of <0.05 for the Wilcoxon signed-rank test and *p*-values of <0.017 for the Kruskal–Wallis test and Mann–Whitney U test were considered statistically significant. All statistical analyses were performed with IBM SPSS Statistics for Windows, Version 24.0 (IBM Corp., Armonk, NY, USA) and GraphPad Prism for Windows, Version 8.4.3 (GraphPad Software, San Diego, CA, USA).

## 3. Results

### 3.1. Patients and Treatment

Twenty right-sided breast cancer patients and 37 left-sided breast cancer patients were included in this study. Of the latter, 24 patients were treated using the DIBH technique, and 13 patients were treated using CPAP. The cumulative numbers of fractions per location and treatment technique were as follows: right-sided breast cancer = 413, pre-treatment CBCT and 361, intra-fractional CBCT; left-sided breast cancer and use of DIBH = 561, pre-treatment and 484, intra-fractional CBCT; and left-sided breast cancer with the use of CPAP = 232, pre-treatment CBCT and 222, intra-fractional CBCT.

The beam angle for the treatment of right-sided breast cancer ranged from 155° to 172° counterclockwise. The starting point of the beam was between 50° and 60°, while the stopping point was between −130° and −150°. For left-sided breast cancer, the beam angle ranged from 169° to 190° clockwise. The starting point was between −45° and −50°, while the stopping point was between 124° and 140°.

### 3.2. Pre-Treatment and Intra-Fractional CBCT

Table 1 summarizes the range of motion (ROM) in centimeters by location and RT technique according to the x-, y-, and z-axes, as well as the absolute difference. For all locations and treatment techniques, larger ROMs were recorded for pre-treatment CBCT scan than for the corresponding intra-fractional CBCT scan. All comparisons of the absolute differences between pre-treatment CBCT and intra-fractional CBCT scan were statistically significant (*p <* 0.001). Therefore, the magnitude of the positional change is likely to be smaller on intra-fractional CBCT than on inter-fractional CBCT, regardless of location or the technique used.

**Figure 2:**
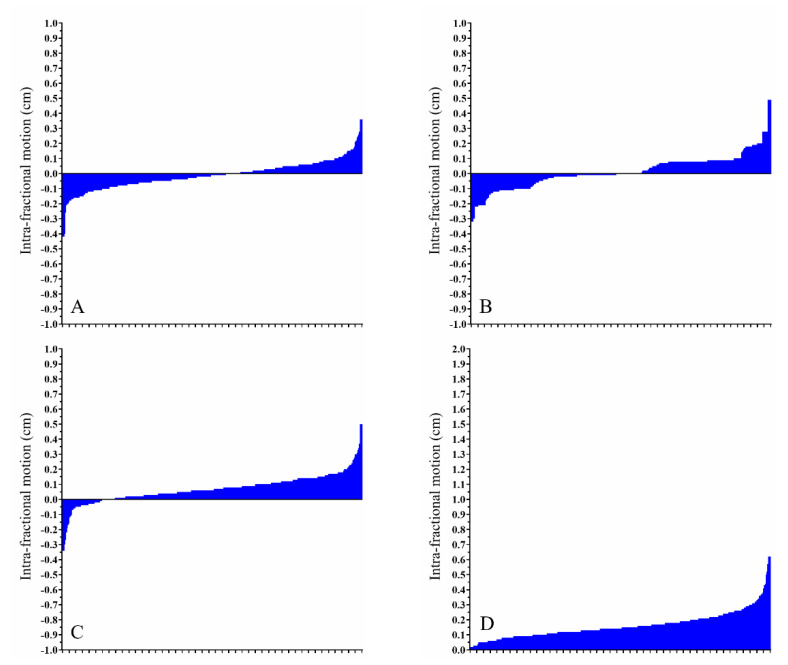
Intra-fractional range of motion for right-sided breast cancer patients. Waterfall plots depicting the intra-fractional positional deviation regarding movements on the (**A**) *x*-axis, (**B**) *y*-axis, and (**C**) *z*-axis and the (**D**) absolute difference.

**Figure 3:**
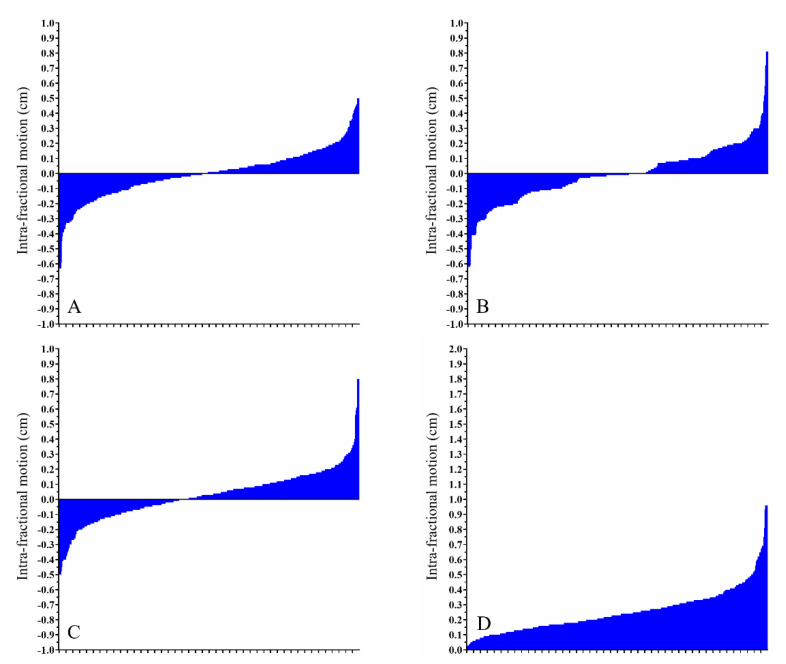
Intra-fractional range of motion for left-sided breast cancer patients using deep-inspiration breath hold technique. Waterfall plots depicting the intra-fractional positional deviations regarding movements on the (**A**) *x*-axis, (**B**) *y*-axis, and (**C**) *z*-axis and the (**D**) absolute difference.

**Figure 4:**
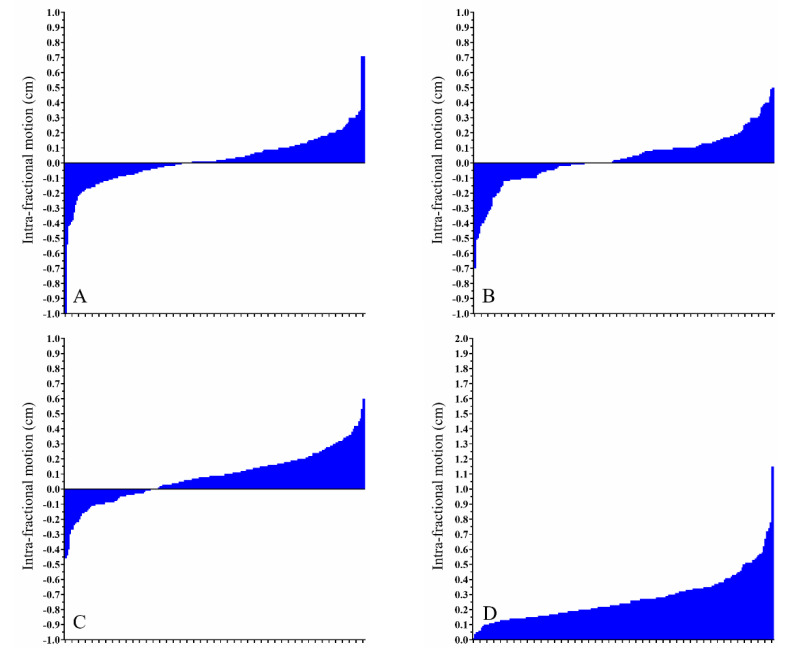
Intra-fractional range of motion for left-sided breast cancer patients using continuous positive airway pressure. Waterfall plots depicting the intra-fractional positional deviation regarding movements on the (**A**) *x*-axis, (**B**) *y*-axis, and (**C**) *z*-axis and the (**D**) absolute difference.

However, even though intra-fractional movement was less than its inter-fractional counterpart, there remain a few exceptions regarding significant positional changes. Figure 2A–D summarizes the intra-fractional ROM in right-sided breast cancer patients. Figure 3A–D displays the intra-fractional ROM in left-sided breast cancer patients treated using the DIBH technique. Figure 4A–D shows the intra-fractional ROM in left-sided breast cancer patients treated using CPAP. The means and standard deviations of intra-fractional ROM were 0.16 cm and 0.09 for the right breast, 0.25 cm and 0.14 for the left breast with the use of DIBH, and 0.27 cm and 0.15 for the left breast with the use of CPAP. Significant differences were found between left-sided breast cancer patients treated using the DIBH technique and right-sided breast cancer patients (*p* < 0.001) and between left-sided breast cancer patients treated using CPAP and right-sided breast cancer patients (*p* < 0.001); however, no statistically significant difference was observed between left-sided breast cancer patients treated using the DIBH technique versus left-sided breast cancer patients treated using CPAP (*p =* 0.094).

### 3.3. Dose-Volume Histogram Analysis

**Figure 5:**
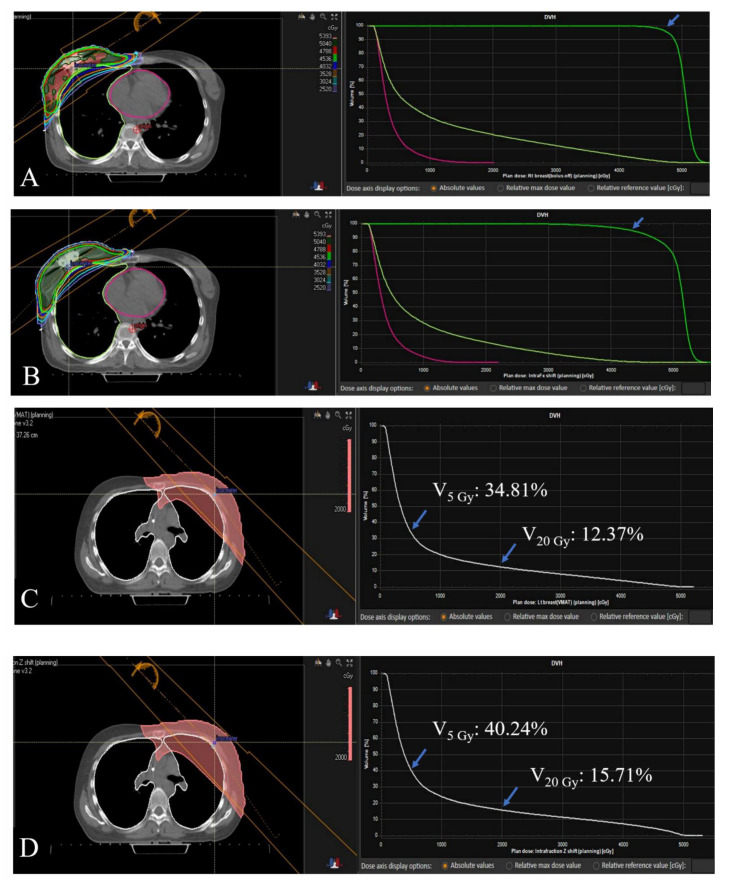
Modification of the plan reflecting the intra-fractional movement resulted in deviation of some key dosimetric parameters in selected cases. In figure (**A**), the dark green and red areas indicate the volumes receiving 100% and 95% of the prescribed dose, respectively. No dark green or red area is shown in figure (**B**). Blue arrows indicate V_95% of the prescription dose_ in figure (**A**,**B**), with respective values of 95.83% and 87.41%. In another case, the lung volume receiving 20 Gy is shown by the pink-colored area (**C**) and is enlarged in figure (**D**), suggesting unnecessary exposure of the normal lung to radiation.

Table 2 summarizes the DVH parameters for the original plan and the modified plan reflecting intra-fractional movements of 10 selected patients. Although these cases were unusual due to their large variations in movement, notable differences were reported in target volume coverage and (increased) radiation doses to OARs. As described in the Methods, 90% was the minimum dose coverage to the target area in terms of D_95% of the PTV_ and V_95% of the prescription dose._ According to the modified plan, seven patients did not meet the minimum requirement, and the target coverage in these cases was deemed insufficient according to our institutional criteria. Patient 1 and Patient 3, whose treatment plans had been considered to be ideal in the original settings, demonstrated a downshift toward the acceptable range. In one extreme case of left-sided breast cancer treated using the DIBH technique, Patient 5 experienced a large decrease in D_95% of the PTV_ from 94.03% to 80.45%. Patients 4 and 7 experienced a more than 20-fold increase in the V_25 Gy_ to the heart in modified plans compared to that in the original plans. Figure 5 displays the exemplary cases of insufficient dose coverage to the target volume and a case of increased dose to the lung.

## 4. Discussion

This study showed the extent of pre-treatment and intra-fractional movement among breast cancer patients receiving IMRT. Our study results suggest that the inter-fractional variation is greater than the intra-fractional variation, thereby strengthening the argument that image-guided verification is necessary before each fraction of treatment. However, intra-fractional movement should not be neglected as the positional deviation during RT could result in suboptimal radiation delivery. This phenomenon was more prominent in left-sided breast cancer than in right-sided breast cancer, according to the results of the current study.

This phenomenon may have been due to the effect of the additional techniques used to avoid normal organ toxicity. The DIBH technique requires patients to hold their breath in a deep inspirational state to inflate the lungs. However, this technique could be physically demanding for some patients [12]. The repetition of this possibly strenuous task of breath holding could lead to extensive internal organ movement. The question concerning the DIBH technique is whether its ability to increase the distance between OARs and the RT target is offset by an unacceptable ROM for the target volume. Although there is a paucity of data, a few studies have reported acceptable ROM of the heart periphery during DIBH [13], and the reproducibility of the breast area has been confirmed by surface imaging [14]. These outcomes are in concordance with the results of our study. We showed a median variation of 0.00 cm, −0.01 cm, 0.03 cm in the x-, y-, and z-axes, respectively. Another method used for normal organ sparing in left-sided breast radiation is CPAP. A study reported the effect of CPAP in left-sided breast cancer patients preparing for RT, showing an increase in lung volume by 60% and a decrease in heart volume by 12% [15]. These CPAP effects are beneficial in preventing radiation-induced toxicity in the heart and the lungs; however, its effect on internal movements during a treatment session is questionable. In our study, those treated with CPAP showed median intra-fractional movements of 0.01 cm, 0.03 cm, and 0.09 cm in the x-, y-, and z-axes, respectively; these values were mostly within the acceptable ROMs. However, both patients treated using the DIBH and CPAP techniques demonstrated wider intra-fractional ROMs than free-breathing right-sided breast cancer patients. Considering the commonly cited side effects of CPAP that cause poor compliance, such as rhinitis, nasal congestion, and aerophagia [16], the discomfort induced by CPAP may contribute to larger ROMs during RT.

The introduction of IMRT and SBRT in the field of radiation therapy opens up a new era of RT with endless possibilities. In this study, we specifically used VMAT which could provide conformal dose distribution in a short beam-on time by adjusting gantry speed, dose rate, and multi-leaf collimator [17]. The pace at which innovations develop is faster than ever. Concurrently with the improvement of RT techniques, there have been developments that reduce RT volumes, and high-dose per fraction regimens can target many primary or metastatic tumor sites [18]. This trend also applies to breast cancer [19], which has a long history of treatment with radiation. For selected ductal carcinoma in situ or early-stage breast cancer patients, PBI using external beam radiotherapy is now becoming an attractive alternative to whole-breast irradiation (WBI) with non-inferior oncologic outcomes [20,21]. Even for WBI, a recent phase III trial showed non-inferior local tumor control with 26 Gy in 5 fractions compared to 40 Gy in 15 fractions [22]. These trials could be characterized by extreme hypofractionation with a larger dose per fraction. This suggests a transition of treatment duration from the conventional 7 weeks to less than 2 weeks. Unlike conventional RT regimens with multiple fractions that could negate non-systematic error in few fractions, the error in each fraction would have had more significance in these hypofractionated settings. Thus, proper verification before each treatment should be guaranteed in the modern RT era. The most common way to verify the RT target volume and adjacent OARs is image-guided RT with volumetric imaging modalities. Even though portal imaging or kV planar imaging is used more frequently than volumetric imaging for the breast [2], some studies have reported the efficacy of CBCT in reducing the planning target volume with minimal setup uncertainties [23,24]. Therefore, the utilization of volumetric imaging verification in breast cancer patients could become more common in the near future. With this perspective, we examined volumetric CBCT images from breast cancer patients who underwent IMRT not only for pre-treatment verification but also for the measurement of intra-fractional movement.

Even though pre-treatment verification to reduce inter-fractional variation is becoming more common, anatomical information during actual beam delivery is not routinely acquired in clinical settings. However, compared to conventional RT, modern RT requires many sophisticated techniques and utilizes numerous beams, which lead to an increased duration of treatment. Therefore, intra-fractional variation as well as inter-fractional variation could be affected. Long treatment times could induce unnecessary motion at the patient’s end. The concept of intra-fractional verification was investigated in multiple studies regarding various organs. One study about SBRT for lung tumors showed that intra-fractional CBCT revealed a maximal target shift of 5.7, 3.6, and 4.9 mm along the anterior-posterior, left-right, and superior-inferior axes, respectively. This meant that an expansion equal to or greater than 7 mm from the gross tumor volume was enough to deal with intra-fractional movement during SBRT for lung tumors with a delivery time of up to 4 min [5]. Another study using an anthropomorphic thorax phantom that evaluated the efficacy of CBCT scans reconstructed from kV images acquired simultaneously during SBRT delivery showed that these scans could help identify the dominant position of tumors during RT delivery [25]. The prostate is also an organ of interest due to its intra-fractional motion during radiotherapy. Many studies have measured intra-fractional movement by comparing pre-treatment CBCT and immediately post-treatment CBCT scans rather than by acquiring simultaneous images. Those studies showed minimal movement of the target area in various treatment settings. For example, one study revealed a movement of only 0.4 mm of the prostate bed during postoperative or salvage RT [26] and a movement of less than 3 mm in patients receiving IMRT after ^125^I implantation according to fiducial-based imaging [27]. For breast cancer patients, the optical surface image was used frequently to evaluate intra-fractional motion during RT. A study with 104 patients and 2028 fractions of RT using an optical surface scanner demonstrated intra-fractional deviation of less than 5 mm in breast RT [28]. However, unlike surface scanning which can only estimate the exact movement of the skin surface, CBCT has a relative advantage as it can assess not only the skin surface but also the relative position of internal structures such as the heart, lungs, bony structures, and chest wall. Thus, CBCT could provide different outcomes compared to skin surface imaging. We identified a study using pre-treatment CBCT and post-treatment CBCT in patients receiving PBI, which suggested the significant effect of intra-fractional errors even with a 10 mm setup margin [29]. A systematic literature review on intra-fractional motion during RT in breast cancer patients reported that although the range of intra-fractional movement in these patients was mostly less than 5 mm, some individuals showed large variations [30]. Even though the review predominantly included studies investigating two-dimensional tangential images, unlike ours in which CBCT images were acquired simultaneously with actual beam delivery, the results from the review are in concordance with our findings.

In our study, most cases showed acceptable intra-fractional deviation with a median of less than 3 mm in terms of absolute distance from the initial isocenter. However, although it is plausible to believe that intra-fractional movement does not cause severe deviations in the majority of cases, there were cases involving low target volume coverage or an increased dose to OARs. Coinciding with the current trend of hypofractionation and smaller target volumes, insufficient dose coverage, even in a single fraction, could result in nonoptimal treatment outcomes. For instance, the impact of a reduction in D_95% of the PTV_ from 94% to 80% in one fraction during the treatment period, seen in our study, would obviously be larger in patients receiving 25 Gy in 5 fractions than in patients receiving 50.4 Gy in 28 fractions. Adequate set-up margin or prompt adaptive planning according to the result of intra-fractional CBCT might be helpful. Especially in VMAT planning, using virtual bolus is another option because it could compensate for internal positional or anatomic change during the course of radiotherapy with better coverage of the target volume [31]. Additionally, cases involving a more than 20-fold increase in V_25 Gy_ of the heart were reported. According to the widely accepted guideline, it is recommended to keep the V_25 Gy_ of the heart at less than 10% to reduce the cardiac mortality risk to less than 1% [32]. Even though the results of our study did not violate the safety criteria for V_25 Gy_ of the heart, watchful observation for OARs is required during RT planning. Specifically, as mentioned above, left-sided breast cancer patients showed an increased likelihood of larger intra-fractional motion, although outliers were reported for both left- and right-sided breast cancer patients. Similar outcomes were reported earlier with a large isocenter variation that considerable affected dosimetric outcomes in a few cases of left-sided breast cancer patients treated using the DIBH technique [33].

Intra-fractional optimization was not possible with intra-fractional CBCT as the final reconstructed images were available only after the treatment. Nevertheless, given that studies analyzing intra-fractional CBCT images in breast cancer patients are scarce, our research has the relative strength by providing quantitative results about the intra-fractional motion and following changes in dosimetric outcomes in selected cases. The limitations of this study include the substandard quality of intra-fractional CBCT scans compared to that of pre-treatment CBCT scans. As intra-fractional CBCT scans are acquired simultaneously with the delivery of treatment beams, beam scattering and subsequent worsening of image quality are unavoidable [34]. However, it is still possible to discriminate significant landmark organs such as the lung, heart, and thoracic wall on intra-fractional CBCT. Considering their possible use in marker-less positional verification and tumor tracking in lung cancer patients [35,36], the quality of intra-fractional CBCT images seems to be acceptable in clinical settings. Moreover, further refinement in CBCT image quality was achieved by utilizing binary moving-blocker-based scatter correction [37]. Another limitation was that an extra dose was delivered to the patient for acquiring intra-fractional CBCT images. However, compared to the high RT dose used for treatment, the additional dose was minimal. Additionally, it is arguable that the set-up margin of our institution is rather too small, and a bigger set-up margin could resolve issues related to the movement of the target and OARs. Still, given that Asian women tend to have denser and smaller breasts compared to the Western population [38,39], a small set-up margin was preferable to avoid skin toxicity.

## 5. Conclusions

In conclusion, this study reported quantitative measurements of inter-fractional and intra-fractional errors in breast cancer patients receiving RT. Pre-treatment CBCT showed larger variations than intra-fractional CBCT, and the range of intra-fractional motion was likely to be bigger in left-sided breast cancer patients using DIBH or CPAP. Even though the intra-fractional ROM was minimal in most cases, few cases showed huge variations that could significantly affect the goal of RT. Therefore, based on the data provided above, the intra-fractional movement might be taken into consideration in specific circumstances when it could be substantial, and even a single non-systematic error could negatively affect treatment outcomes.

## Figures and Tables

**Table 1:** Range of motion according to treatment location and radiotherapy technique.

	*x*-Axis	*y*-Axis	*z*-Axis	Absolute Difference
Treatment Location and Radiotherapy Technique	Median (Range)	Median (Range)	Median (Range)	Median (Range)
Rt. breast pre-treatment CBCT, Free-breathing (cm)	−0.07 (−0.78–0.64)	0.14 (−2.92–1.30)	0.29 (−1.69–0.94)	0.53 (0.06–2.98)
Rt. breast intra-fractional CBCT, Free-breathing (cm)	−0.01 (−0.42–0.36)	0.00 (−0.32–0.49)	0.06 (−0.34–0.50)	0.14 (0.00–0.62)
Lt. breast pre-treatment CBCT, DIBH (cm)	0.12 (−1.32–0.84)	-0.03 (−1.86–4.26)	0.29 (−1.97–1.83)	0.66 (0.08–4.41)
Lt. breast intra-fractional CBCT, DIBH (cm)	0.00 (−0.63–0.50)	-0.01 (−0.62–0.81)	0.03 (−0.50–0.80)	0.23 (0.02–0.96)
Lt. breast pre-treatment CBCT, CPAP (cm)	0.03 (−1.83–1.41)	0.00 (−3.72–3.18)	0.07 (−1.92–1.50)	0.69 (0.04–3.80)
Lt. breast intra-fractional CBCT, CPAP (cm)	0.01 (−1.11–0.71)	0.03 (−0.70–0.50)	0.09 (−0.46–0.60)	0.24 (0.00–1.15)

CBCT, cone-beam computed tomography; CPAP, continuous positive airway pressure; DIBH, deep-inspiration breath hold.

**Table 2:** Summary of changes in dose-volume histogram values between the original plan and modified plan.

Patients (Location; Technique)	D_95% of the PTV_ (% of Prescribed Dose)	V_95% of the prescribed dose_ (% of the PTV)	Mean Lung Dose (Gy)	Ipsilateral Lung V_5 Gy_ (%)	Ipsilateral Lung V_20 Gy_ (%)	Heart D_0.2 cc_ (Gy)	Heart V_25 Gy_ (%)
Patient 1 (Right-sided)							
Original	96.1	96.9	9.53	56.07	12.15	24.49	0.51
Modified	94.9	95	9.05	55.33	10.9	22.21	0.48
Patient 2 (Right-sided)							
Original	95.47	95.83	8.23	45.27	11.2	14.15	0
Modified	86.03	87.41	7.01	42.52	8	12.86	0
Patient 3 (Right-sided)							
Original	98.02	99	11.05	73.1	13.44	23.61	0.62
Modified	93.53	94	9.82	71.38	9.78	21.57	0.18
Patient 4 (Left-sided; DIBH)							
Original	96.77	97.28	8.01	34.81	12.37	21.39	0.15
Modified	86.88	88.41	9.68	40.24	15.71	31.62	3.32
Patient 5 (Left-sided; DIBH)							
Original	94.03	93.54	6.27	38.31	5.95	11.71	0
Modified	80.45	81.55	5.75	38.2	3.85	10.87	0
Patient 6 (Left-sided; DIBH)							
Original	94.76	94.88	8.43	57.51	8.84	19.02	0.01
Modified	86.41	83.64	10.05	61.73	12.64	22.35	0.36
Patient 7 (Left-sided; DIBH)							
Original	91.67	90.02	9.67	62.38	11.57	26.35	1.48
Modified	87.26	88.07	9.75	64.93	11.53	32.41	3.87
Patient 8 (Left-sided; CPAP)							
Original	95.75	96.25	7.89	43.06	11.25	14.39	0
Modified	90.3	89.02	6.61	39.66	8.09	13.16	0
Patient 9 (Left-sided; CPAP)							
Original	93.19	92.43	7.83	41.44	11.27	13.74	0
Modified	90.52	90.62	9.39	45.37	14.92	14.75	0.02
Patient10(Left-sided; CPAP)							
Original	93.77	93.4	8	41.66	11.55	16.09	0
Modified	90.44	89.6	7.08	39.18	9.27	15.52	0

CPAP, continuous positive airway pressure; PTV, planning target volume; DIBH, deep-inspiration breath hold.

## Data Availability

The data presented in this study are available on request from the corresponding author. The data are not publicly available due to IRB restrictions.
